# Creating National Weights for a Patient-level Longitudinal Database

**DOI:** 10.36469/9828

**Published:** 2016-03-23

**Authors:** Onur Baser, Li Wang, Jon Maguire

**Affiliations:** 1 Center for Innovation & Outcomes Research, Department of Surgery, Columbia University, New York, NY; STATinMED Research, New York, NY; 2 STATinMED Research, Plano, TX; 3 Senior Director, Optum, New York, NY

**Keywords:** national representation, raking, propensity score matching

## Abstract

**Objective:** To create a nationally-representative estimate from longitudinal data by controlling for sociodemographic factors and health status.

**Method:** The Agency for Healthcare Research and Quality’s (AHRQ) Medicare Expenditures Panel Survey (MEPS) was used as the basis for adjustment methodology. MEPS is a data source representing health insurance coverage cost and utilization, and comprises several large-scale surveys of families, individuals, employers, and health care providers. Using these data, we created subset populations. We then used multivariate logistic regression to construct demographics and case-mix-based weights, which were applied to create a population sample that is similar to the national population. The weight was derived using the inverse probability of the weighting approach, as well as a raking mechanism. We compared the results with the projected number of persons in the US population in the same categories to examine the validity of the weights.

**Results:** The following variables were used in the logistic regression: Age group, gender, race, location, income level and health status (Charlson Comorbidity Index scores and chronic condition diagnosis). Relative to MEPS data, patients included in the private insurance data were more likely to be male, older, to have a chronic condition, and to be white (p=0.0000). Adjusted weighted values for patients in the commercial group ranged from 15.47 to 36.36 (median: 16.91). Commercial insurance and MEPS data populations were similar in terms of their socioeconomic and clinical categories. As an outcomes measure, the predicted annual number of patients with prescription claims from private insurance data was 6 963 034. The annual number of statin users were predicted as 6 709 438 using weighted MEPS data.

**Conclusion:** National projections of large-scale patient longitudinal databases require adjustment utilizing demographic factors and case-mix differences related to health status.

## INTRODUCTION

When researchers ask for a nationally-representative sample, they mean that the population of interest is equivalent to the entire population of the country in question, and the sample should reflect this in its structure. A nationally-representative sample should match the number of men versus women according to national proportions, and the percentage in each age group or region should match the population. In outcomes research, matching based on health status is also crucial.

The first step for any sampling project is to identify the "universe" or "target population" of subjects for which inferences are desired.[1] Most data in outcomes research (i.e. commercial insurance claims data, regional trial datasets) may contain subsets of the target population in proportions that do not match the ratios of those groups in the population itself. Figure 1 presents the regional distribution of a commercial insurance claims data population relative to the US national population. The data is underrepresented in the West and Midwest United States, but is overrepresented in the South and Northeast. Conversely, in the Medicare advantage population, the data is overrepresentative in West and underrepresentative in the others.

**Figure 1.** Regional Distribution of Commercial Insurance Claims Data relative to National Data Affiliated Health Plans, Commercially Insured Population with Medical and Pharmacy Benefits[2,*,†]

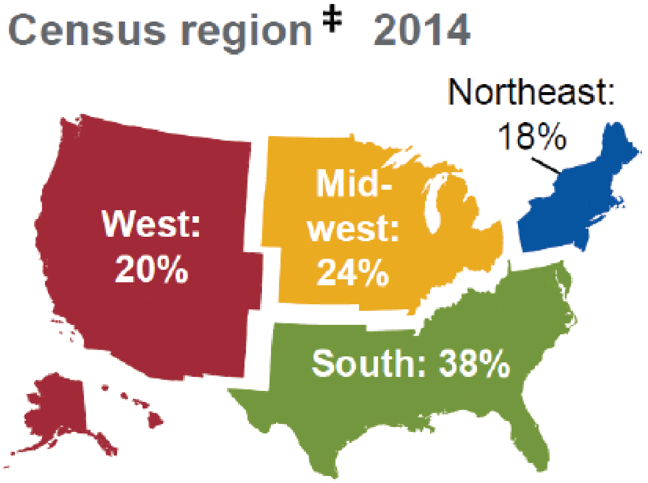

| | West | Mid-west | South | North-east |
|---|---|---|---|---|
| Commercial[§] | 2.8M | 3.3M | 5.3M | 2.5M |
| % of Commercial | 20% | 24% | 38% | 18% |
| US Est.[||] | 23% | 23% | 35% | 19% |

*Statistics current through December 31, 2014.
†Members counted here may also be counted in other administrative segments because they can move between segments over time. Members do not appear in multiple administrative segments during the same period of time.
‡Includes members enrolled at any time in 2014.
§Excludes members where attribute is unknown.
||Source of estimate of US commercially insured population: US Census Bureau, Current Population Survey, Annual Social and Economic Supplement, 2014. Health insurance questions asked respondents about their status in the previous year (2013). Accessed at http://www.census.gov/cps/data/cpstablecreator.html.

Figure 2 shows the distribution by age group. Among certain age groups, there are significant differences between the two data populations.

**Figure 2.** Age Distribution Comparison between Commercial and National Databases[2,*,†]

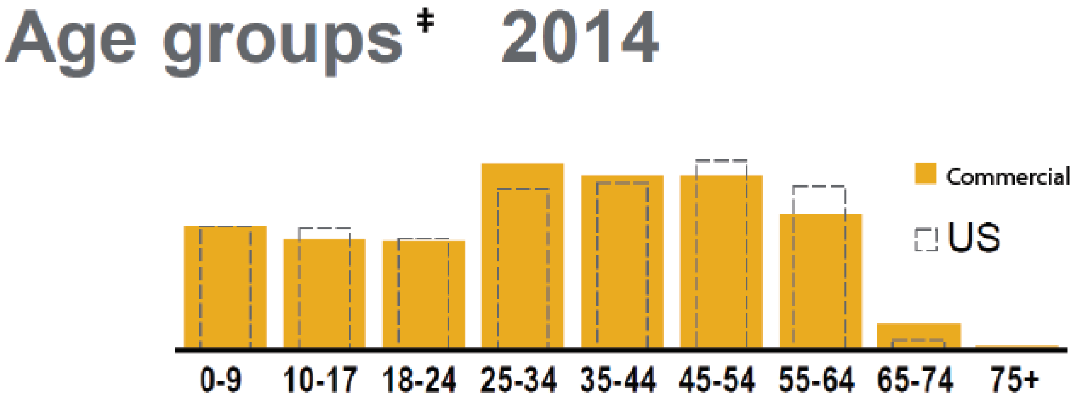

| | 0-9 | 10-17 | 18-24 | 25-34 | 35-44 | 45-54 | 55-64 | 65-74 | 75+ |
|---|---|---|---|---|---|---|---|---|---|
| Commercial§ | 1.6M | 1.5M | 1.4M | 2.5M | 2.3M | 2.3M | 1.8M | 0.4M | 0.1M |
| % of Commercial | 12% | 11% | 10% | 18% | 17% | 17% | 13% | 3% | 1% |
| US Est. ‖ | 12% | 12% | 11% | 15% | 16% | 18% | 16% | 1% | 0% |

*Statistics current through December 31, 2014.
†Members counted here may also be counted in other administrative segments because they can move between segments over time. Members do not appear in multiple administrative segments during the same period of time.
‡Includes members enrolled at any time in 2014.
§Excludes members where attribute is unknown.
‖Source of estimate of US commercially insured population: US Census Bureau, Current Population Survey, Annual Social and Economic Supplement, 2014. Health insurance questions asked respondents about their status in the previous year (2013). Accessed at http://www.census.gov/cps/data/cpstablecreator.html.

In such situations, one can often improve the relationship between the sample and the population by creating weights based on specified characteristics that agree with the corresponding totals for the population.[3]

One way to create weights is to match each cell defined by the cross-classification of categorical variables to control data which is usually chosen from a national data source. However, to make an argument that the sample represents the national population, adjustment for case-mix differences is necessary, since simple adjustment for demographics would not be adequate. For non-demographic measures (i.e. comorbid and chronic conditions) the sample should also match the population.

We analyzed the difference between a commercial insurance dataset and the household component of the Agency for Healthcare Research and Quality (AHRQ) Medicare Expenditures Panel Survey (MEPS) in terms of demographic factors and health status. We attempted to improve the estimates from the commercial dataset by creating weights for each case patient, so that marginal totals of the adjusted weights agree with the corresponding totals (demographic and non-demographic) for the population according to specified characteristics. This operation is known as raking — an analogy for raking is the process of smoothing the soil in a garden plot by working it back and forth with a rake in two perpendicular directions. The statistical procedure is discussed in detail by Bishop, et al.[5] and Deming.[6]

## METHODS

A commercial multi-source patient longitudinal database and the household component of the MEPS data provided the two data sources for this study.

The commercial dataset has a proprietary research database containing claims and enrollment data dating back to 2000 with the ability to link patient and physician survey data to pharmacy and medical claims, medical record data, socioeconomic measures, and clinical laboratory results. For 2005, data relating to approximately 14 million individuals with both medical and pharmacy benefit coverage are available. Underlying information is geographically diverse across the United States and is frequently updated. The household component of MEPS collects data from a sample of families and individuals in selected communities across the United States drawn from a nationally-representative sub-sample of households that participated in the prior year's National Health Interview Survey. For 2005, data relating to 32 320 individuals are available.

The socioeconomic characteristics included in the model were: Head of the household age, female patient percentage, race, US geographic region, and income level.

We derived two variables to capture general health status of the member. First, Charlson Comorbidity Index (CCI) scores were generated to capture the level and burden of comorbidity. The most commonly used index in health outcomes studies is the CCI, which assigns a weight ranging from 1 to 6 according to disease severity for 19 conditions.[7] The CCI contents and weighting scheme are based on Cox proportional hazards modeling.[8] The weights for each condition are summed, and a score is assigned to each patient. The original index was developed in an inpatient setting, using medical review to predict the risk of mortality. The index has since adopted several weights, some of which allow outpatient diagnoses to contribute to the score.[9,10] Regardless of the version, the CCI has practically insignificant effects in predicting health care utility and indices.[11,12]

Secondly, we created an indicator variable to represent patients with chronic conditions. This variable was derived by convening two physician panels to review all medical conditions reported by the survey sample.

Our model proceeds in three steps using inverse probability weighting and raking strategy. Initial sampling weights were calculated as the inverse of the probability of selection. These weights play a pivotal role in design-based inferences to yield estimates that are intended to be unbiased and consistent. Finally the initial weights were adjusted so that the marginal total of adjusted weight on specified characteristics agrees with the corresponding totals for the population.

The basic raking algorithm with two variables such as age and gender can be described as follows:

Let $\{\eta_i : i = 1,...,n\}$ denote our initial weight estimated from the second step for sample size $n$ from the population. In a post-stratification that has $J$ rows and $K$ columns, let $n_{jk}$ be the sum of the $\eta_i$ in cell $(j,k)$.

Initial row and column totals of the initial weights and population numbers are defined as $\eta_{j+}$, $\eta_{+k}$, $P_{j+}$ and $P_{+k}$ respectively.

The first three steps of the algorithm are $1: w[1] = \eta_{j,k}; 2: w[2] = w[1]*(\frac{P_{j+}}{\eta_{j+}})$ for each $k$ within each $j$; and $3:\ w[3] = w[2]*\frac{P_{+k}}{\eta_{+k}}$ or each $j$ within each $k$. In the iteration process, both row and column weights are adjusted.

By adjusting for eligibility status at each month and quarter, we derived monthly and quarterly weights as well as annual weights.

To validate the initial weights, the MEPS sample was randomly categorized into two groups: Training subsample and test sub-sample. Weights were calculated using the training sub-sample. The weighted means were estimated for each confounder from the commercial data. These values were then compared with the mean of same variables from the MEPS test sub-sample.

Final weights (after raking) were validated by comparing the results with those for the projected number of people in the US population in each category.

## RESULTS

Table 1 shows the results of logistic regression to identify the differences between the commercial and MEPS populations, in terms of socioeconomic and clinical factors. Patients in the commercial data population were more likely to be male, older, and white. The probability of being in the commercial data sample was close to four times higher for patients diagnosed with chronic conditions.

**Table 1.** Socioeconomic and Clinical Factor Differences in Commercial and MEPS Data

| | | 95% CI | |
|---|---|---|---|
| **Variables** | **Odds Ratio** | **Lower** | **Upper** |
| **Age in Years** | | | |
| Under 5 | | Reference | |
| 5-17 | 2.08 | 1.88 | 2.31 |
| 18-44 | 1.71 | 1.55 | 1.97 |
| 45-64 | 1.63 | 1.47 | 1.8 |
| **Sex** | 1.88 | | |
| Male | | Reference | |
| Female | 0.94 | 0.9 | 0.99 |
| **Race/ethnicity** | | | |
| White | | Reference | |
| Hispanic or Latino | 0.55 | 0.52 | 0.59 |
| Black-single race/not Hispanic | 0.39 | 0.36 Table 2. Summary of the Annual, Monthly and Quarterly Weights created to Project the US Population from a Commercial Dataset | 0.41 |
| Other | 4.02 | 3.5 | 4.61 |
| Asian/Pacific Islander-single race/not Hispanic | 0.46 | 0.41 | 0.5 |
| **Poverty Status** | | | |
| Low income | | Reference | |
| High income | 11.16 | 10.53 | 11.82 |
| **Census Region** | | | |
| Northeast | | Reference | |
| Midwest | 1.15 | 1.07 | 1.23 |
| South | 2.28 | 2.13 | 2.43 |
| West | 1.15 | 1.07 | 1.23 |
| **Chronic** | | | |
| Non-Chronic | | Reference | |
| Chronic | 4.38 | 4.1 | 4.68 |
| Charlson Comorbidity Index Score | 0.61 | 0.55 | 0.68 |

CI: confidence interval

Table 2 shows the summary of annual, monthly, and quarterly weights after raking. These weights were used to project the US population from the commercial data population.

**Table 2.** Summary of the Annual, Monthly and Quarterly Weights created to Project the US Population from a Commercial Dataset

| Variable | Minimum | Maximum | Mean | Median |
|---|---|---|---|---|
| Annual | 15.47 | 36.36 | 16.99 | 16.91 |
| Month 1 | 15.14 | 35.59 | 16.63 | 16.55 |
| Month 2 | 15.17 | 35.65 | 16.66 | 16.58 |
| Month 3 | 15.27 | 35.90 | 16.78 | 16.70 |
| Month 4 | 15.35 | 36.07 | 16.86 | 16.78 |
| Month 5 | 15.43 | 36.27 | 16.95 | 16.87 |
| Month 6 | 15.47 | 36.36 | 17.00 | 16.91 |
| Month 7 | 15.59 | 36.65 | 17.13 | 17.05 |
| Month 8 | 15.63 | 36.73 | 17.17 | 17.08 |
| Month 9 | 15.63 | 36.74 | 17.17 | 17.09 |
| Month 10 | 15.64 | 36.77 | 17.19 | 17.10 |
| Month 11 | 15.65 | 36.78 | 17.19 | 17.11 |
| Month 12 | 15.64 | 36.77 | 17.19 | 17.10 |
| Quarter 1 | 15.23 | 35.80 | 16.74 | 16.65 |
| Quarter 2 | 15.35 | 36.09 | 16.87 | 16.79 |
| Quarter 3 | 15.53 | 36.50 | 17.06 | 16.98 |
| Quarter 4 | 15.75 | 37.03 | 17.31 | 17.22 |

Table 3 shows the projected number of people in the US population after applying the weights for both data sources. The differences in the predictions for each category (socioeconomic and clinical) were negligible.

**Table 3.** Projected Number of People in the US Population using Commercial and MEPS Data Populations

| | | Commercial | |
|---|---|---|---|
| | | US Population (MEPS Data) | US Population (Commercial Data) |
| Population Characteristics | | Population (in thousands) | Population (in thousands) |
| Total | | 183 033 | 183 033 |
| **Age (years)** | | | |
| | Under 5 | 11 377 | 11 350 |
| | 5-17 | 35 159 | 35 168 |
| | 18-44 | 78 181 | 78 109 |
| | 45-64 | 58 315 | 58 326 |
| **Sex** | | | |
| | Male | 91 322 | 91 323 |
| | Female | 91 710 | 91 710 |
| **Race/Ethnicity** | | | |
| | Hispanic or Latino | 18 092 | 18 085 |
| | Black-single race/not Hispanic | 18 593 | 18 598 |
| | White, other, 2 or more races/not Hispanic | 137 742 | 137 746 |
| | Asian/Pacific Islander-single race/not Hispanic | 8606 | 8603 |
| **Poverty status** | | | |
| | Low income | 27 653 | 27 656 |
| | High income | 155 379 | 155 377 |
| **US Census Region** | | | |
| | Northeast | 35 213 | 35 204 |
| | Midwest | 43 376 | 43 369 |
| | South | 63 204 | 63 213 |
| | West | 41 239 | 41 245 |
| **Chronic Disease** | | | |
| | Non-Chronic | 148 759 | 150 315 |
| | Chronic | 34 263 | 32 718 |

MEPS: Medicare Expenditures Panel Survey

We also created weights to predict outcomes measures: Annual statin users and number of statin prescriptions. Projected annual statin prescriptions for commercial population from commercial data were 53 412 217. The projections from quarterly weights are shown in Figure 3. Predicted annual statin users for the commercial health care insurance population from commercial data were 6 963 034. The number predicted using MEPS data with MEPS weight was 6 709 438. Projections from quarterly weights are shown in Figure 4.

**Figure 3.** Projected Total Annual Number of Statin Prescriptions using Quarterly Weights

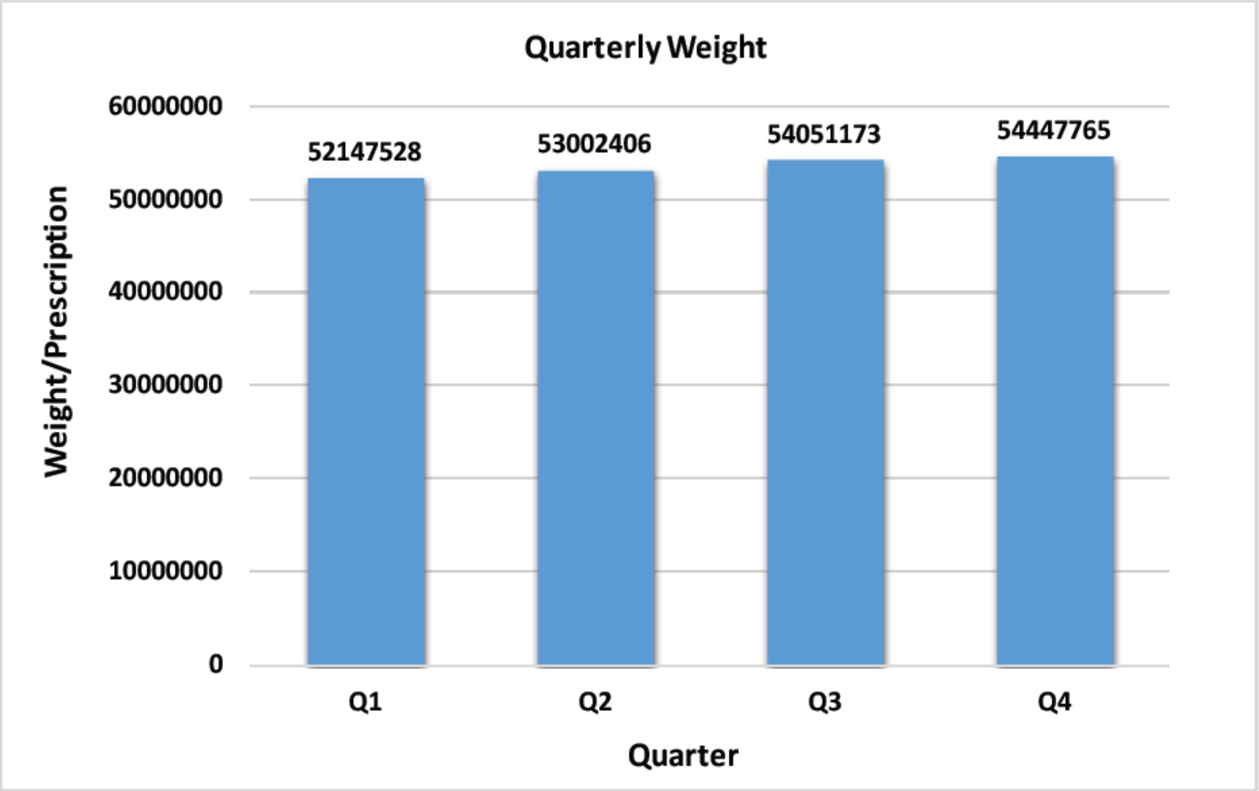

**Figure 4.** Summary of the Annual, Monthly and Quarterly Weights created to Project the US Population from the a Commercial Patient Population

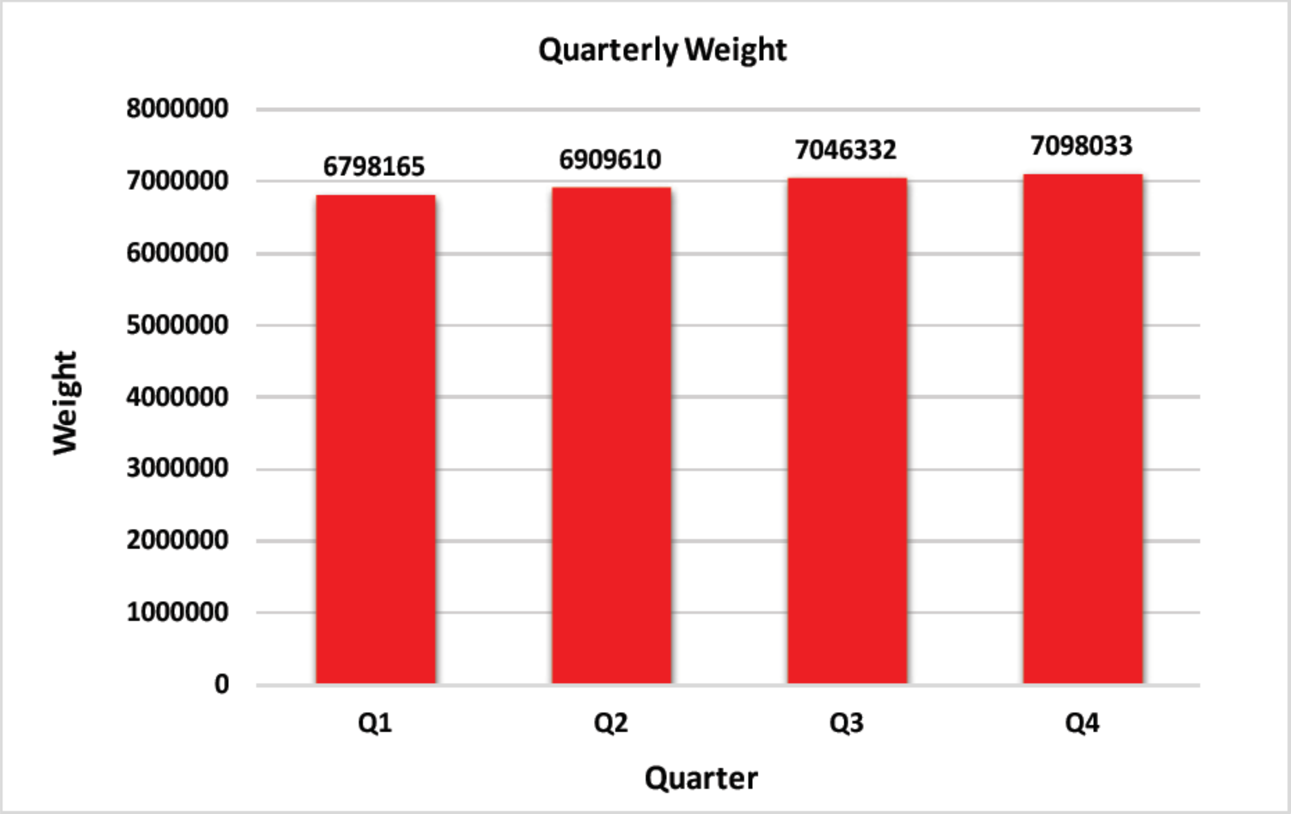

## CONCLUSION

National projections of large-scale patient longitudinal databases require adjustment utilizing demographic factors and case-mix differences related to health status. The differences between commercial data and the household component of the MEPS, in terms of demographic factors and health status, were examined. We incorporated these differences into our model to calculate weights and examined the validity of the created weights. The created weights successfully balanced the population in terms of comorbid and chronic conditions, and demographic factors.

## CONFLICT OF INTEREST DECLARATION

The authors declare they have no conflicts of interest. This study was not funded.
